# The extravascular implantable cardioverter‐defibrillator: The pivotal study plan

**DOI:** 10.1111/jce.15190

**Published:** 2021-08-05

**Authors:** Ian Crozier, David O'Donnell, Lucas Boersma, Francis Murgatroyd, Jaimie Manlucu, Bradley P. Knight, Ulrika Maria Birgersdotter‐Green, Christophe Leclercq, Amy Thompson, Robert Sawchuk, Sarah Willey, Christopher Wiggenhorn, Paul Friedman

**Affiliations:** ^1^ Department of Cardiology Christchurch Hospital Christchurch New Zealand; ^2^ Department of Cardiology Austin Health Heidelberg Victoria Australia; ^3^ Department of Cardiology St. Antonius Hospital Nieuwegein and Amsterdam UMC Amsterdam Netherlands; ^4^ Department of Cardiology King's College Hospital London UK; ^5^ Division of Cardiology London Health Sciences Centre London Ontario Canada; ^6^ Division of Cardiology Northwestern University Chicago Illinois USA; ^7^ Division of Cardiology University of California San Diego San Diego California USA; ^8^ Department of Cardiology CHU de Rennes—Hôpital Pontchaillou France Rennes France; ^9^ Department of Cardiac Rhythm Medtronic plc Mounds View Minnesota USA; ^10^ Department of Cardiovascular Medicine Mayo Clinic Rochester Minnesota USA

**Keywords:** anterior mediastinum, defibrillation, extravascular, ICD, subcutaneous, substernal

## Abstract

**Background:**

Transvenous implantable cardioverter defibrillators (TV ICD) provide life‐saving therapy for millions of patients worldwide. However, they are susceptible to several potential short‐ and long‐ term complications including cardiac perforation and pneumothorax, lead dislodgement, venous obstruction, and infection. The extravascular ICD system's novel design and substernal implant approach avoids the risks associated with TV ICDs while still providing pacing features and similar generator size to TV ICDs.

**Study Design:**

The EV ICD pivotal study is a prospective, multicenter, single‐arm, nonrandomized, premarket clinical study designed to examine the safety and acute efficacy of the system. This study will enroll up to 400 patients with a Class I or IIa indication for implantation of an ICD. Implanted subjects will be followed up to approximately 3.5 years, depending on when the patient is enrolled.

**Objective:**

The clinical trial is designed to demonstrate safety and effectiveness of the EV ICD system in human use. The safety endpoint is freedom from major complications, while the efficacy endpoint is defibrillation success. Both endpoints will be assessed against prespecified criteria. Additionally, this study will evaluate antitachycardia pacing performance, electrical performance, extracardiac pacing sensation, asystole pacing, appropriate and inappropriate shocks, as well as a summary of adverse events.

**Conclusion:**

The EV ICD pivotal study is designed to provide clear evidence addressing the safety and efficacy performance of the EV ICD System.

## 1 INTRODUCTION

Transvenous implantable cardioverter defibrillators (TV ICD) are effective protective devices against sudden arrhythmic death. However, they are limited by various implantation risks including vascular injury, cardiac perforation and pneumothorax.^1^ Venous obstruction, lead failure and infection are longer term complications that may occur years after the original implant.^2^, ^3^ Device infections can be serious, with the potential for bacteremia or endocarditis due to the physical conduit between the TV ICD subcutaneous pocket and the ICD lead extending into the heart and systemic circulation. For these, and various other reasons, transvenous leads may ultimately require extraction, a relatively high‐risk procedure associated with the potential for significant morbidity and mortality.^4^, ^5^, ^6^ Therefore, alternatives to TV ICDs are desired to reduce morbidity associated with endovascular and/or endocardial lead placement. The Extravascular ICD System with its substernal lead placement may offer an alternative to transvenous systems while positioning a lead close to the heart. The EV ICD has been purposely designed to provide life‐saving defibrillator therapy, whilst addressing some of the concerns of conventional transvenous and subcutaneous implantable defibrillators.

The subcutaneous defibrillator (S‐ICD), implanted extravascularly, was previously developed to avoid the vascular risks of TV ICDs.^7^, ^8^ In a recent comparison with TV ICDs, S‐ICD device complications tended to be fewer whilst offering similar protection from sudden arrhythmic death and until recently the inappropriate shock rate tended to be higher (with the first generation without the new algorithm to discriminate SVT from VT).^9^, ^10^ However, since the S‐ICD lead is placed above the sternum and some distance from the myocardium, the energy required for defibrillation is high relative to TV ICD, necessitating a large device (60 cc) with compromised longevity (projected 7.3 years with normal use).^11^ Furthermore, because of the distance between the electrical lead and the heart, the only pacing possible is limited to 30 s of postshock support.^7^ Moreover ATP is not possible with this device.

A number of case reports have described placing leads in the retrosternal space to achieve defibrillation. In 2007, Tung et al.^12^ reported implant of a “transvenous” lead into the retrosternal space from a superior (manubrial) approach in three patients with venous occlusion or refusing transvenous hardware and achieved successful defibrillation with a 10‐Joule (J) safety margin using standard transvenous ICD device energy outputs. Subsequently, multiple examples of successful “subcutaneous” lead placement in the retrosternal space have been reported.^13^, ^14^, ^15^, ^16^, ^17^

The EV ICD System, a product of a development program initiated in 2012 by Medtronic, is comprised of a pulse generator implanted subcutaneously over the serratus anterior in the left midaxillary line and a high voltage lead in the substernal space. A series of animal studies and proof‐of‐concept studies confirmed a lead could be positioned in the anterior mediastinal space, allowing for defibrillation with energies lower than those required by the S‐ICD, and demonstrating feasibility of ventricular pacing from this site.^18^, ^19^

The first human clinical feasibility study, The acute substernal defibrillation (ASD) study, showed substernal defibrillation was feasible with the energy available in current TV ICDs, with defibrillation successful in 13 of 14 subjects at 35 J.^20^ In the second human clinical feasibility study, the Substernal Pacing Acute Clinical Evaluation (SPACE), 26 subjects underwent pacing evaluation, using a substernal decapolar catheter placed temporarily at the time of planned cardiac procedures. It showed that pacing is possible in nearly all patients from the substernal location.^21^, ^22^ The third human clinical feasibility study, The Acute Extravascular Defibrillation, Pacing and Electrogram Study (ASD2), further assessed pacing thresholds and defibrillation efficacy via substernal therapy delivery in a larger cohort with an EV ICD lead designed for sensing, pacing and defibrillation in the substernal space. In 79 patients, the median lead implantation time was 12.0 ± 9.0 min. Ventricular pacing was successful in at least 1 vector in 76 of 78 patients (97.4%), and a 30‐J shock successfully terminated 104 of 128 episodes (81.3%) of ventricular fibrillation in 69 patients.^23^

Subsequently, 21 permanent EV ICD implants were performed in 2018 in the first‐in‐human chronic EV ICD Pilot study.^24^ In the Pilot study, 20 patients had the device successfully implanted, of which 18 patients (90%) had induced ventricular arrhythmias successfully terminated by the EV ICD system. Pacing capture was achieved in 95% of study patients at implant. There were no intraprocedural complications, perhaps resulting from measures to facilitate a safe procedure, including a mandatory hands‐on training program for implanters, cardiothoracic surgical proctoring, blunt dissection to access the substernal space, and use of fluoroscopic guidance in two views (lateral and AP). This study confirmed the potential of the EV ICD System to be implanted safely and provide effective defibrillator therapy, thus leading to the start of the EV ICD Pivotal trial.

## 2 METHODS

### 2.1 Study design

The EV ICD Pivotal study is a prospective, multicenter, single‐arm, nonrandomized, premarket clinical study, approved by local regulatory and ethics committees and conducted in accordance with the ethical principles of the Declaration of Helsinki at up to 60 sites worldwide, intended to include ANZ (Australia and New Zealand), Canada, EMEA (Europe, Middle East, and Africa), Hong Kong, Japan, and the United States. Up to 400 subject enrollments will allow at least 292 subjects to complete defibrillation testing of the implanted system. The study commenced in September 2019, and enrollments are expected to be completed mid‐2021. At the time of manuscript submission, over 215 subjects have been enrolled (ClinicalTrials.gov, Registration No. NCT04060680). All relevant abbreviations are detailed in Table 1.

**Table 1:** Abbreviations and acronyms

AE: adverse event
ANZ: Australia/New Zealand
AP: anterior posterior
ASD: the acute substernal defibrillation study
ASD2: acute extravascular defibrillation, pacing and electrogram study
ATP: antitachycardia pacing
CEC: clinical events committee
CT: computerized tomography
DFT: defibrillation threshold testing
EMEA: Europe, Middle East, and Africa
ERC: episode review committee
EV: extravascular
ICD: implantable cardioverter‐defibrillator
J: Joule
OPC: objective performance criteria
PHD: pre‐hospital discharge
SPACE: substernal pacing acute clinical evaluation
SSVA: sustained shockable ventricular arrhythmias
SVT: supraventricular tachycardia
TV: transvenous
VF: ventricular fibrillation
MRI: magnetic resonance imaging

### 2.2 The EV ICD system

**Figure 1:**
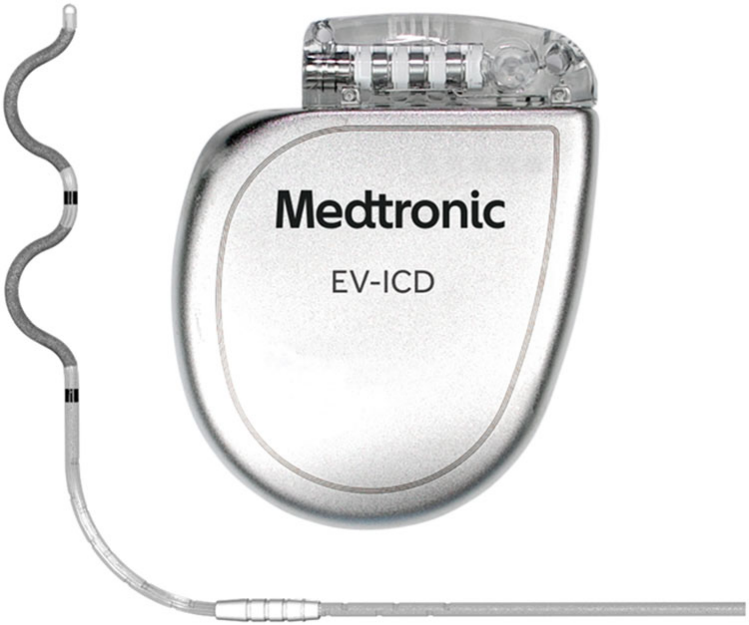
EV ICD System. Extravascular implantable cardioverter defibrillator (EV ICD) and EV ICD quadripolar lead with passive fixation

The EV ICD System (Figure 1) was designed for the substernal space; the device is a modified and enhanced version of the Evera magnetic resonance imaging (MRI) System, the size of a transvenous defibrillator (33 cm^3^) and capable of delivering up to 40 J of defibrillation energy. The EV ICD lead uses an epsilon‐shaped design for passive fixation within the substernal space, and to intentionally orient the available electrodes to the left to optimize sensing and pacing therapies and the coils to the right to facilitate defibrillation therapy. There are two pace/sense electrodes and two defibrillation coil segments that are electrically coupled during defibrillation to form an 8 cm defibrillation coil. There are three sensing and three pacing vectors available in the EV ICD system.^23^

### 2.3 Implantation procedure

**Figure 2:**
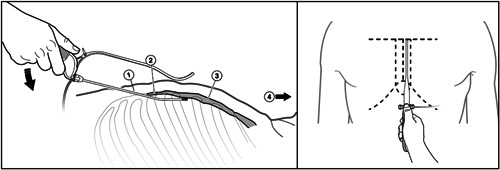
EV ICD implant overview. Left panel: Lateral view. Tunneling rod tip at the top of the cardiac silhouette (1, Tunneling Rod, 2, Xiphisternal Junction, 3, Sternum, 4, Head). Right panel: Exterior view: tunneling rod tip at the top of the cardiac silhouette. EV, extravascular; ICD, implantable cardioverter defibrillator

**Figure 3:**
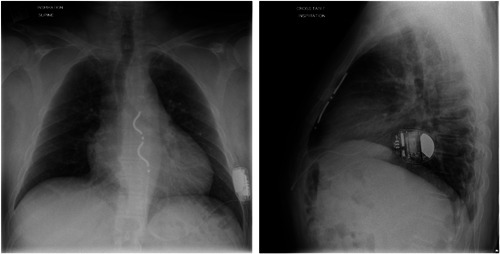
EV ICD System in situ. Anteroposterior (left) and lateral (right) fluoroscopic images of fully implanted system (Image from EV ICD Pilot study patient). EV, extravascular; ICD, implantable cardioverter defibrillator

All implanting investigators will complete a formal training program on the recommended implant technique for the EV ICD system to establish skills in safe access and tunneling within the substernal space. Cardiac Surgeons are encouraged to attend the formal training program with the primary investigator but are also able to be trained on‐site if necessary, before the first case. Implants will be performed by the trained investigator with cardiothoracic surgical backup available. General anesthesia is recommended, and external defibrillation pads will be placed outside the surgical field for rescue defibrillation if required. Chest X‐rays (AP and lateral) will be collected at baseline for all patients, and computerized tomography scans or MRI will be recommended for the first three implants; imaging may help in preprocedure planning and assessment of anatomy. To access the substernal space, an incision (approximately 3 cm) will be made between the inferior point of the xiphoid and the left costal margin. Blunt dissection is then performed beyond the rectus fascia and through the diaphragmatic attachments. The dedicated implant tunneling tool will be placed within a peel‐away introducer sheath and then be introduced and advanced utilizing lateral fluoroscopy to ensure the tip of the tunneling tool is in close proximity to, or direct contact with, the posterior surface of the sternum to avoid cardiac injury. The tunneling path will extend to the upper border of the cardiac silhouette as marked by the lower margin of the carina using AP fluoroscopy, and between the midline and left sternal margin (Figure 2). The EV ICD lead will be inserted into the substernal space via the peel‐away introducer sheath once the tunneling tool is removed. After deployment of the lead, acute sensing measurements will be collected with the requirement of R‐wave amplitudes ≥1 mV and P wave ≤0.2 mV. If need be, the lead can be repositioned to achieve the sensing targets. The lead will then be anchored to the fascia in the subxiphoid incision. The proximal portion of the lead will be tunneled to a left lateral subcutaneous device pocket near the midaxillary line. The device will be placed against the fascia and sutured within the pocket. Following hemostasis, the incisions will be closed using standard closure techniques (Figure 3: EV ICD System in Situ). Programming recommendations and requirements at discharge are provided (Appendix I).

### 2.4 Study population

Patients with a Class I or IIa indication for implantation of an ICD according to the ACC/AHA/HRS/ESC Guidelines^25^, ^26^; who are at least 18 years of age; are geographically stable and willing and able to complete the study procedures and follow‐up will be enrolled after providing written informed consent. Patients will be excluded from the first generation of the system if they have indications for bradycardia pacing or cardiac resynchronization therapy. Additionally, key study exclusions included patients with an existing system, leads, or neurostimulator or other chronically implanted device which delivers current; patients with medical interventions which might increase surgical risk to patient during the tunneling procedure (e.g., prior or planned sternotomy, prior chest radiotherapy), patients with medical conditions or abnormalities which might increase procedure risk, infection risk, or risk of potential comorbidities which might impact the evaluation of the system during a clinical study (e.g., pectus excavatum, decompensated heart failure), patients who might be more vulnerable to potential increased risk during the evaluation of the clinical study defibrillation protocol (e.g., hemodynamic instability, current intracardiac left atrial or left ventricular thrombus, left ventricular ejection fraction <20%) or contraindication for temporary suspension of oral/systemic anticoagulation.

### 2.5 Study objectives and endpoints

The main purpose of the study is to test the safety and acute efficacy of the EV ICD System. The primary safety objective is to demonstrate the freedom from major complications related to the EV ICD System and/or procedure up to 6‐month postimplant, defined as a subject's first occurrence of a major complication related to the EV ICD System and/or procedure as adjudicated by an independent Clinical Events Committee. The study will meet the prespecified objective performance criterion (OPC) if the freedom from major complications exceeds 79% (i.e., the lower bound of a two‐sided 95% confidence interval exceeds 79%). (Appendix II. Safety endpoint definition).

The primary efficacy objective is to demonstrate defibrillation efficacy at implant of the EV ICD system. Sustained shockable ventricular arrhythmias (SSVA) (e.g., ventricular fibrillation, polymorphic ventricular tachycardia, rapid ventricular tachycardia) will be induced at implant using burst induction (20 Hz), T‐shock (up to 20 J), or other methods at the discretion of the investigator. Testing is deemed successful if a ≥10 J defibrillation safety margin is present with the 40 J system, and defined as termination of an SSVA with either a single 20 J shock or on two consecutive episodes of SSVA at 30 J shock in the final configuration (Appendix III. Defibrillation Protocol at Implant). If the patient is successfully defibrillated at 20 J, defibrillation efficacy will be assessed at 15 J. The study protocol incorporates shock polarity reversal and revision of lead and/or pulse generator position in the event of initial failure. If defibrillation success is not achieved, the EV ICD system will be explanted. The study will meet the prespecified OPC if the defibrillation success at implant is greater than 88% (i.e., the lower bound of a two‐sided 95% confidence interval exceeds 88%).

Ancillary objectives will include characterization of appropriate and inappropriate shocks; electrical performance (pacing capture thresholds, pacing impedance, sensing amplitudes) over time; extracardiac pacing sensation; asystole pacing; ATP performance with spontaneous arrhythmias and summary of adverse events. In a subgroup of up to 34 patients that prospectively consent to this, chronic defibrillation efficacy will be evaluated at 6‐month postimplant; subjects will be induced to produce up to two episodes of VF, with detection and termination at either 30 or 40 J regarded as successful.

### 2.6 Follow‐up plan

**Figure 4:**
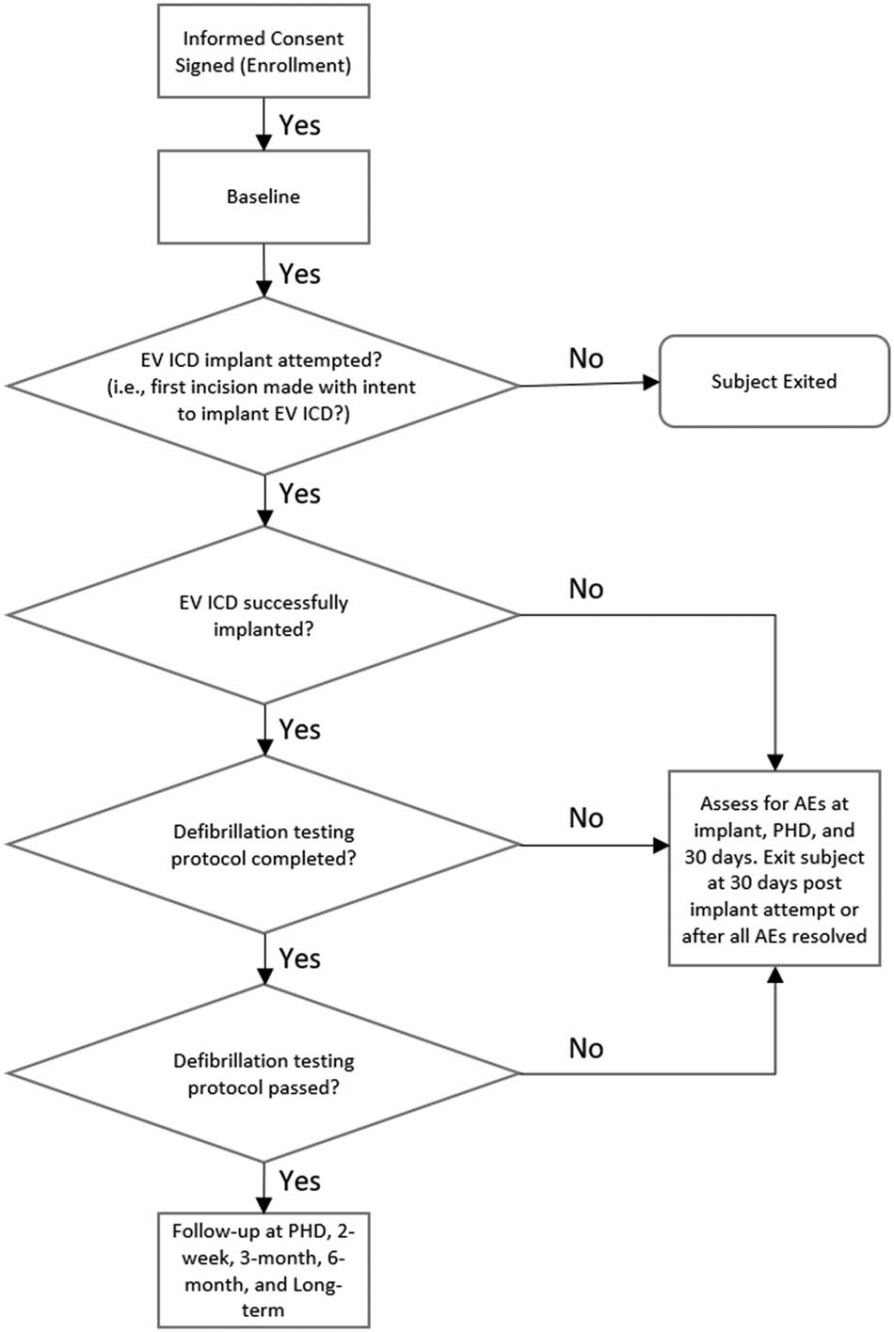
Study overview. AE, adverse event; PHD, pre‐hospital discharge

Patients will be evaluated during follow up at 2 weeks, 3 months, 6 months and then every 6 months until completion of the trial (Figure 4, study overview). Assessments will include device interrogation; electrical testing (sensing, impedance, and pacing tests); adverse event rate and type; device deficiency rate and type; healthcare utilizations; and medications. Chest radiographs (anteroposterior and lateral) will be obtained before discharge and at 6 months.

### 2.7 Study organization

Study oversight includes a Steering Committee with members from various geographies, responsible for advising on study design and execution. An independent Clinical Events Committee (CEC) will review and adjudicate all system‐ and procedure‐related events, as well as death classifications. An Episode Review Committee, which includes independent physicians, will evaluate and adjudicate device‐treated ventricular episodes and appropriateness of therapy. An independent data monitoring committee will review incidents and trends of adverse events and make recommendations to Medtronic and/or the Steering Committee regarding study conduct and subject safety.

### 2.8 Statistical methods

The analysis of the primary safety objective will include all subjects with an implant attempt. The endpoint consists of major complications related to the EV ICD System and/or procedure within 182 days of implant attempt, as determined by an independent CEC (Appendix II. Safety endpoint definition). Subjects not experiencing an event will be censored at their last point of contact. The 182‐day freedom from major complication rate will be estimated using the Kaplan–Meier method, along with a two‐sided 95% confidence interval based on a log–log transformation. If the lower bound of the confidence interval exceeds 79%, the primary safety objective will be met.

In estimating statistical power for the safety objective, the occurrence of major complications was modeled with a Weibull distribution, assuming an event‐free rate of 90% at 1 month and 86% at 6 months. Attrition due to exit or death was also assumed to follow a Weibull distribution, at a rate of 9% at 1 month and 16% through 1‐year postimplant. Using these assumptions, the outcome of the trial was simulated 10 000 times. Each simulated sample included 292 subjects, with a simulated time to safety endpoint and time to attrition for each subject. Using these data, the 182‐day Kaplan–Meier freedom from major complication rate and a 95% confidence interval was calculated for each simulation of the trial. The results of the simulation determined that a sample size of 292 subjects undergoing an implant attempt allowed for 90% power for this objective.

For the primary efficacy objective, each subject who completes the defibrillation protocol will be categorized as a success or failure. A two‐sided exact binomial 95% confidence interval will be calculated for the proportion of successes, and the efficacy objective will be met if the lower bound of the confidence interval exceeds 88%. In calculating the sample size for this objective, it was assumed that the true success rate is 93.5%. Using the statistical software package PASS 2008, it was determined that 292 subjects completing the defibrillation protocol were required to achieve 90% power. To further account for subjects who enroll in the study but exit before an implant attempt, up to 400 subjects may be enrolled.

## 3 DISCUSSION

The EV ICD System uses a novel substernal lead implant location designed to avoid the risks associated with transvenous defibrillators and the limitations of the S‐ICD. The study is designed to evaluate the safety and efficacy of the EV ICD system as well as features unavailable in the S‐ICD system, such as ATP pacing and pause prevention pacing, with no requirement for preimplant sensing evaluation screening. The study will also characterize appropriate and inappropriate shocks, which is of interest as the EV ICD lead does not have direct myocardial contact; as smaller R‐waves have been reported previously for the S‐ICD, the EV ICD system will be similarly evaluated for T‐wave and noncardiac sensing observations.^27^

The success criterion (i.e., OPC) of 79% was used to evaluate system‐related complications in the pivotal trial for the S‐ICD. By comparison, the EV ICD pivotal study is evaluating both system‐ and procedure‐related complications using an OPC of 0.79 at 6 months to establish safety.

Similarly, the OPC threshold of 88% was used in the pivotal trial for S‐ICD as the criterion for evaluating termination of induced ventricular rhythms at implant, and the EV ICD pivotal study uses the same criterion to evaluate efficacy. In a retrospective analysis of trends and in‐hospital outcomes associated with early adoption of the S‐ICD compared to single‐ and dual‐chamber transvenous ICD implants, DFT testing efficacy among 2791 patients was shown to be 92.7% when a 15‐J safety margin was used for S‐ICD.^28^ Additional studies and subanalyses of defibrillation testing performance for S‐ICD show evidence of defibrillation testing performance of less than the OPC of 88%, further justifying the clinical relevance of such a threshold.^29^, ^30^

In conclusion, the EV ICD System has similar capabilities to a single‐chamber transvenous ICD system while avoiding leads in the heart and vasculature. Therefore, the EV ICD system may become the preferred option for many patients indicated for a single chamber ICD in the future. Compared to the current market‐released nontransvenous subcutaneous ICDs, the EV ICD system includes a smaller device that uses less defibrillation energy which may result in longer battery life but with the additional capabilities to deliver pacing therapies such as ATP and pause prevention pacing from a single device. Although the EV‐ICD requires substernal tunnelling with the potential for cardiac injury during implantation, this device has the potential to be the optimal defibrillator in many patient groups, especially young patients in whom long term defibrillator therapy is envisaged.

During the trial we have required cardiothoracic surgical backup; however, if the safety goal of the trial is achieved with a low rate of cardiac injury or need for surgical intervention, this may not be required for clinical implants by experienced implanters in the future. The EV ICD Pivotal study will demonstrate the efficacy and safety of the EV ICD System: a single‐chamber extravascular ICD system with the lead implanted substernally.

## CONFLICT OF INTERESTS

Dr. Crozier is a consultant for and receives research support and fellow support from Medtronic plc and grants from Boston Scientific Corp. Dr. O'Donnell has received research support and/or served as consultant to Abbott and Medtronic. Prof. Boersma is a consultant for Medtronic, Boston Scientific, Philips, Acutus, and Adagio and his institution receives grant support from Medtronic and Boston Scientific. Dr. Murgatroyd is a consultant for Medtronic, Inc. In the last 3 years he has also consulted for Abbott, Inc, and Boston Scientific, Inc., and his department has received research support from Abbott, Inc, Boston Scientific, Inc. and Medtronic, Inc. Dr. Manlucu is a consultant for and receives research and fellowship support from Medtronic. She also has received research support from and/or served as consultant to Abbott, Boston Scientific and Baylis Medical. Dr. Knight is a consultant for and receives research support and fellowship support from Medtronic. He also has received research support from and/or served as consultant to Abbott, Boston Scientific, Biotronik, CVRx, Baylis, and Philips. Dr. Birgersdotter‐Green receives honoraria from Medtronic and BCS, receives honorarium and research grants from Abbott, and is on an advisory board for Biotronik. Prof. Leclercq receives speaker fees from Medtronic, Abbott, Biotronik, Boston, Microport and research grants from Biotronik, Medtronic. Ms. Thompson, Mr. Sawchuk, Ms. Willey, and Mr. Wiggenhorn are employees of Medtronic. Dr. Friedman has received research support and/or served as consultant to Abbott, Medtronic, Boston Scientific, and Leadex, with all proceeds going to Mayo Clinic. He has licensed intellectual property to NeoChord, Preventice, AliveCor, Anumana, Champion Medical, Marani Health, and MediCool.

## Data Availability

As this is a study design manuscript there are no data available to share. Data sharing not applicable—no new data available at this stage.

## Appendix I Pre‐hospital discharge (PHD) Programming Requirements and Recommendations for patients who have undergone implantation with an EV ICD System

**Table:**

Required programming		Recommended programming	
Initial NID for VF Therapies	Minimum 30/40	VF Sensing	Per implant testing results (most sensitive setting)
All Rx	40 J	VF Sensing if PHD Troubleshooting is required	Per PHD testing results (most sensitive setting)
Pause Prevention	Monitor, 5 s	Post Shock Pacing	Per physician recommendation; 4 V margin minimum
		ATP	At physician discretion; 4 V margin minimum

## Appendix II safety endpoint definitions

For an adverse event to meet the endpoint, the event must have occurred within 182 days (inclusive) of the EV ICD System implant and be adjudicated by the Clinical Events Committee as being a major complication related (causal relationship) to the EV ICD System and/or procedure. Major complications are those complications resulting in:

- Death
- Permanent loss of defibrillation function (specifically shock) due to mechanical or electrical dysfunction of the device
- Hospitalization
- Prolongation of an existing hospitalization by at least 48 h
- System revision (reposition, replacement, explant)

## Appendix III defibrillation protocol at implant

Defibrillation testing protocol at implant procedure, outlining (Panel A): the process of SSVA induction and defibrillation testing and (Panel B): troubleshooting recommendations in the event of failure of first episode. Under each episode number, the boxes indicate the testing sequences dependent on the outcome of the previous defibrillation test.

**Table:**

Panel A:

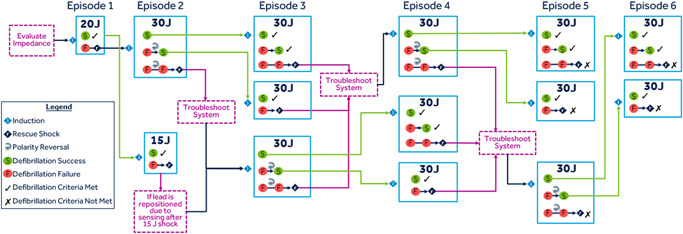

Panel B: Troubleshooting. Prior to inducing and throughout the defibrillation protocol, it is recommended to consider the following to improve defibrillation outcome or troubleshooting in the event of failure of first episode(s):
Check for/resolve pneumothorax Check for/resolve high impedance values Check for/resolve air in tunnel (e.g., fluoroscopy) Check for/resolve air in pocket (e.g., flush with saline or antibiotic wash, massage air out of pocket) Check for/resolve gastric bubbles (gas) Press on device pocket during testing Perform defibrillation during held end tidal expiration (end expiration apnea) If all of the above measures are exhausted, evaluate the position of the EV ICD device and the EV ICD Lead. If required, consider repositioning of EV ICD generator or EV ICD lead.^1^ Allow time (e.g., next day or during the admission) to minimize transient factors affecting defibrillation success
^1^Lead revision is not permitted if the subject has resumed anticoagulation. Post‐procedure anticoagulation should be resumed as soon as possible unless clinically contraindicated (e.g., effusion observed) in subjects who have had atrial fibrillation for ≥48 h in duration prior to the implant procedure and who convert to sinus rhythm during defibrillation testing to diminish the risk of periprocedural stroke.
